# Ghanaian media coverage of violence against women and girls: implications for health promotion

**DOI:** 10.1186/s12905-018-0621-1

**Published:** 2018-08-24

**Authors:** Ebenezer Owusu-Addo, Sally B. Owusu-Addo, Ernestina F. Antoh, Yaw A. Sarpong, Kwaku Obeng-Okrah, Grace K. Annan

**Affiliations:** 1 Bureau of Integrated Rural Development (BIRD), Kwame Nkrumah University of Science and Technology (KNUST), Kumasi, Ghana; 2 Ejisu-Juaben Municipal Health Directorate, Ghana Health Service, Ejisu, Ghana; 3 Health Promotion Department, Ghana Health Service, Accra, Ghana

**Keywords:** Health promotion, Frame analysis, Ghana, Media, Violence against women and girls

## Abstract

**Background:**

Violence against women and girls (VAWG) is an important public health issue. Framing studies indicate that how the news media cover public health issues is critical for designing effective health promotion interventions. Notwithstanding this, there is little research particularly in low-and middle-income country context examining how the news media frame VAWG. This paper examines news coverage of VAWG in Ghana, and the implications of this for health promotion.

**Methods:**

This study used frame analysis as the methodological framework in examining how VAWG in Ghana is represented by the media. Qualitative content analysis approach to frame analysis was performed on 48 news articles which constituted the unit of analysis.

**Results:**

The findings indicate that media framing of VAWG was episodic in nature as the acts of violence perpetrated against women and girls were presented as individual cases without reference to the wider social contexts within which they occurred. Similarly, victim blaming language was largely used in the news articles. In framing VAWG as an individual incident and women as helpless victims, the media fail to shape society’s perception of VAWG as a social and public health issue.

**Conclusions:**

For the media in Ghana to contribute to the prevention of VAWG, there is the need for news coverage to focus on social construction of the issue, and also raise awareness about support services available to victims.

## Background

### Overview of violence against women and girls (VAWG)

Globally, VAWG is a major public health threat for victims and society at large. A recent report of WHO with London School of Hygiene and Tropical Medicine and Medical Research Council [1] indicates that worldwide, about 35% of women have experienced either physical and/or sexual intimate partner violence or non-partner sexual violence.

In Ghana, though records are not up-to-date, in 2011, the Domestic Violence and Victim Support Unit of the Ghana Police Service (DOVVSU) [2] reported 12,906 cases of various forms of VAWG including physical assault, sexual assault and murder inflicted by intimate partners. Similarly, a total of 17,655 domestic violence cases, 1296 cases of defilement^1^ and 335 rape cases were reported in 2014 [3]. Aside from sexual violence and domestic abuse which are widespread in Ghana [4], other forms of VAWG in the society include outmoded cultural practices such as widowhood rites, forced marriages, childhood betrothal, child marriages, female genital mutilation, and *trokosi* in which young virgin girls are placed in servitude to fetish shrines to atone for offences committed by family members [5]. The incriminating of older women, often widows, who are targets of witchcraft accusations is also a major form of gender violence in Ghanaian society as such women are often banished to neglected witch sanctuaries [6]. Also, although attitudes and practices are changing due to globalisation and influences of formal education, the typical Ghanaian family is still a patriarchal institution. In the domestic realm, men consider it a marital right to control and discipline their wives resulting in frequent intimate partner violence against women [7].

The public health implications of VAWG are enormous. VAWG affects all aspects of a woman’s life – her sovereignty, her productivity, and her capacity to take responsibility for herself and her children, and subsequently also her overall health status and quality of life [1]. Violence against women has also been identified as an important cause of morbidity from physical, sexual and reproductive health outcomes, and it is also associated with known risk factors for poor health, such as alcohol and drug use, smoking and unsafe sex [8]. Studies from Ghana indicate that violence against women has harmful effects on taking pregnancy to full term and the health of unborn babies [9].

Given the impacts and high prevalence of VAWG, a recent series in the 2014 edition of the Lancet which was devoted to VAWG made a strong call for a concerted effort of various stakeholders to prevent all forms of VAWG [8, 10, 11] The report of the Expert Group Meeting on Prevention of VAWG identified the media as one of the key stakeholders that could help fight VAWG [12]. That is, the news media are well positioned to play an important role in the construction of social problems like VAWG [13]. Studies on framing effects indicate that whether and how the media cover VAWG, including the frequency of reportage, labelling, and information included or omitted could make a difference in how society views VAWG and how it can be prevented [14].

### News media framing of VAWG

Entman [15] provides a critical perspective on how media framing informs audience interpretation of events through the process of selection and salience. He notes that, to frame is to “select some aspects of a perceived reality and make them more salient in a communicating text, in such a way as to promote a particular problem definition, causal interpretation, moral evaluation, and/or treatment recommendation” (p. 52). By highlighting aspects of information about an issue, frames elevate them in salience, often via placement or repetition, or association with culturally familiar symbols. At the same time, frames omit and/or obscure other aspects of the issue [15]. Entman’s definition resonates with Gitlin’s [16] who defines media frames as “persistent patterns of cognition, interpretation, and presentation, of selection, emphasis, and exclusion, by which symbol-handlers routinely organize discourse” (p. 7). Gitlin’s definition places emphasis on routine organisation (selection, emphasis and exclusion) which goes beyond a given story to meaningfully structure the social world. In this case, frames have power to transform otherwise nebulous reality into a meaningful structure. Sieffe [17] further explains that it is through repetitive presentation of news frames to the public that gradually shapes the way an issue is seen. The central premise of news framing, therefore, is that, frames will, by highlighting certain aspects of events, lead audience members’ thoughts about the event in predictable ways, to logical conclusions. Framing thus, both influences which events appear in the news and how they are reported.

The literature on framing discusses two categories of frames: episodic and thematic framing. Episodic framing has been identified as the predominant form of news media coverage where issues are shown as particular instances, and are individualistic in nature, without recourse to broader context of the issue – thus tending to blame the victim. Thematic framing, however, puts issues into their broader perspectives and the social context, for example by using incidence and prevalence rates, social explanations of the issue or a discussion on the broader causes of the problem – thus eliciting societal/structural support [18]. For instance, Iyengar [19] analyses television news frames that focused on specific events (episodic frames) compared to those that put events in a wider context (thematic frames) for a number of political issues, and concluded that “exposure to episodic news makes viewers less likely to hold public officials accountable for the existence of some problem and also less likely to hold them responsible for alleviating it” (p. 2–3). Easteal et al.’s [20] study which examined the power of the media to transform the public opinion about violence against women found that the media create its own reality of VAWG by using framing tactics that separate the issue from the wider social context. A number of studies have shown that news media framing can affect what individuals think about risk options, political issues, and social issues [21, 22].

Journalists’ choice of words or language has been found to impact on how VAWG is framed in the news media. A study by Judd and Easteal [23] which examined media portrayal of sexual harassment in Australia, showed that the media relied heavily on broad-brush characterization and suggestive language, which created its own ‘reality’ of sexual harassment. Bullock and Cubert’s [24] study which examined newspaper coverage of domestic violence in Washington State, United States (US) observed that most headlines did not include language that distinguished an intimate partner homicide from a homicide between strangers, thus influencing the public’s understanding of the problem and the participants involved. Further, Gillespie et al.’s [25] study on news media framing of femicide in North Carolina, US, identified six frames (normalizing the event as commonplace, framing the event as an isolated incident, finding some fault in the criminal justice system, victim blaming, moral breakdown by the perpetrator, and minimizing the event by focusing on a broader issue) that did not portray femicide as domestic violence. Other studies have also observed that news media coverage of VAWG often use language that tends to blame the victim [26–28]. Victim blaming language shifts the responsibility for addressing VAWG from society to the individual.

In spite of the news media’s role in shaping the public’s perception of VAWG, there has been a relative lack of studies examining media reportage on VAWG in low-and middle-income country settings. Most studies conducted on this subject have largely focused on high-income country settings such as the US, Australia, United Kingdom (UK) and Canada [14, 21, 24, 25, 28–33]. In response to the relative void in the literature on media coverage of VAWG, and the call for increased role of the media in VAWG prevention, this study focuses on analysing how VAWG in Ghana, a lower-middle income country is framed by the media, and examines the implications of this for health promotion. This study will provide useful lessons and insights to inform global and local policy makers, health promotion practitioners, journalists and other advocates in developing appropriate interventions to strengthen the role of the media in the prevention of VAWG in Ghana.

## Methods

### Design

The methodological tool used in this study is frame analysis. Two approaches are dominant when using framing as a methodological strategy: quantitative content analysis and qualitative content analysis [34]. Different from quantitative content analysis which uses standardised measures to code and compare texts, qualitative content analysis examines and identifies underlying frames from media narratives [35]. News frame analysis, as a form of qualitative content analysis thus allows researchers to progress from studying tangible narratives to developing a conceptual interpretation that helps to further understand those media realities. As argued by Andersson and Lundström [36], media reportage is not a reflection of objective facts, but descriptions with claims and interpretations constructed by journalists. Also, the media do not operate in an ideological vacuum as a number of factors influence news media framing including social norms and values, organisational pressures and constraints, pressures of interest groups, journalistic routines, and ideological or political orientations of journalists’ and media organisations [37]. Qualitative content analysis was chosen for the frame analysis as it aligns with the study aim of exploring how the media frame VAWG in the news. Similarly, the design is appropriate in that there is limited research literature on media coverage of VAWG in Ghana which precludes the use of preconceived categories required in a quantitative content analysis approach to frame analysis.

### Setting

Ghana, a lower-middle income country on the west coast of Africa, is divided into 10 administrative regions and 216 decentralized districts. The 2010 Population and Housing Census report [38] estimates Ghana’s population at 24,658,823 with 50.9% of population living in urban areas. The census further estimates that females constitute 51.2% of the population and males 48.8%, resulting in sex ratio of 95 males to 100 females [38]. With regard to literacy, the 2010 census indicates that roughly 74% of the population aged 11 years and above is literate. The literacy rate for males is 80.2%, whereas that for females is 68.5%. In urban areas, literacy rate is 84.1% while the rate is 62.8% in rural areas [38]. Although English is the official language in the country, the population is ethnically heterogeneous with multilingualism.

Like many other low-and middle-income countries, the health system in Ghana is overly stretched due to inadequate health facilities, lack of equipment and high burden of communicable diseases. There is in place a National Health Insurance Scheme (NHIS) which aims to promote equity in access to health care. However, less than one-half of the population are members of the NHIS. Though a Mental Health Law (Act 846) was passed in 2012, psychiatric care and access to mental health services are limited [39].

Regarding press freedom, Ghana made great strides in history for the freedom of the media when the Criminal Libel Law which restricted press freedom and criminalised free speech since colonial era was repealed in 2001 [40]. This has offered media houses and journalists the opportunity to express their views on issues and activities in the country without fear or intimidation. At present, media proliferation is on the ascendency in the country due to the liberalisation of the landscape.

### Data collection and sampling

The newspapers included in this study were: *The Ghanaian Times, Daily Graphic* and *Daily Guide*. The FM stations were *Peace FM* and *Joy FM*. The newspapers selected for this study are national in character and are published daily except Sundays [41]. *The Ghanaian Times* and *Daily Graphic* are state-owned daily newspapers while *Daily Guide* is a renowned privately owned newspaper [42]. These newspaper outlets are nationally representative and give coverage to a wide range of issues. They are the most highly subscribed newspapers by the general public, government ministries, departments and agencies, academic and research institutions and other local and international organisations operating in the country [41]. *Peace FM* (*peacefmonline.com*) and *Joy FM* (*myjoyonline.com*) are private radio stations with wider coverage across the country and are the most popular, and leading radio stations in Ghana [7]. The websites of these two leading radio stations provide English versions of their news items broadcasted to the general public in the local language. A further search was conducted in the website of the *Ghana News Agency (GNA*) to complement the five media outlets. The media sampled for this study provide the greatest possible representation in Ghana [41].

A systematic approach was used to search for news items covering issues on VAWG in the six selected media outlets from January 2014 to December 2014. This time frame was chosen due to increased reported cases of VAWG (*N* = 19,286) in that year to examine how the media covered the issues^2^. Only news articles published in English^3^ were chosen because of lack of resources to interpret or translate sources in other languages. To be included in the study, the news articles had to report incidence of violence against women or girls in Ghana. News items describing such incidents from overseas were excluded. Trained research assistants were tasked to manually search the three newspapers daily to identify issues captured on VAWG using the inclusion criteria. The first and second authors searched the websites of the two radio stations and the *GNA* for news items on VAWG.

For cases with only one news article, that individual article was selected for inclusion in the analysis. Duplicate reports written by the same reporter were removed by manual checking as in some cases, news items reported in the newspapers were also found in the websites of the radio stations and vice-versa. When a duplicate of a news item was found, the one that captured detailed report on the issue was included in the study.

### Analysis

The approach to the analysis was to analyse each article as a separate phenomenon. Each individual article thus constituted the unit of analysis rather than coding each case collectively. Bullock [14] notes that issues pertaining to frames in media reportage can include the frequency of coverage, labelling, information included and omitted, and episodic or thematic focus. Therefore, using the article rather than the case as the unit of analysis offered the benefit of ensuring more accurate representation of the occurrence and/or representation of the news frames.

Merriam ([43]: 152) observes that the point of departure for analysing documents (in this case the news articles) is to “adopt some system of coding and cataloguing them” as a data management tool. Following this, the analysis relied heavily on ‘readings’ and interpretations of the media texts with the aim of coding the primary news frame. Throughout the analysis process, attention was paid to contextual factors as required in qualitative content analysis and not just the text in its face value. This approach was found helpful to gaining broader picture of the texts written by the journalists.

To facilitate the coding process, all news articles obtained from the newspapers were first transcribed verbatim by research assistants. These transcripts were then added to the news articles obtained from the websites of the radio stations and the *GNA* to form a complete dataset for analysis. The combined transcript was read several times by the first and second authors to identify meaningful units of text at the familiarization stage. This inductive process resulted in the development of a coding sheet with the following contents: basic information of news item such as source of news item, headline, date, nature of violence, perpetrator, cause/reason of violence, action taken against the perpetrator, framing of victim and perpetrator, explanation of violence perpetrated. The coding framework was then systematically applied to all transcripts using a manual approach where shorthand codes were written on both margins of the transcript using coloured highlighting pens to make comparison, both within and between cases. Text that could not be categorised with the initial coding scheme was given a new code. Following this inductive approach, the news frames were derived by sorting out codes into organising and basic frames in each news article. The organising frame is the main theme found in the news article, while the basic frame is a supplementary frame that speaks to the main theme.

## Results

The following results first describe the sample and then second, discuss the two organising frames identified from the news articles: VAWG as a private/individual issue and victim blaming. These two organising frames and their basic frames are supported by direct quotations from the news articles.

### Search results

The media search identified 215 relevant articles from the six media outlets. After applying the inclusion criteria, and removal of duplicates, a total of 48 news articles reporting on 52 cases were included in the analysis. *The Ghanaian Times* and *Myjoyonline.com* reported the highest number of cases relating to VAWG comprising 35% and 25% respectively. Of the 52 cases, 19 cases (37%) were covered by different media outlets. All other cases (63%; *N* = 33) were covered in only one article by a single media outlet. Most of the articles were short, and incident focused as compared to in-depth analysis. Taken as a group, murder cases (6/7) received wider coverage followed by rape (5/8), and defilement (9/21) cases in both the print and electronic media.

As shown in Table 1, more than half (74%) of VAWG cases in the sample took place in urban areas (population of more than 5000) whereas the remaining 26% occurred in rural areas (population less than 5000 [34]. Most of the news articles focused on acts of violence against women (52%; *N* = 27) with the remainder focusing on acts of violence against girls (48%; *N* = 25). With the exception of news articles which focused on reports produced by organisations, all other news articles were descriptive in nature. Among the women who suffered violence, about 80% of them were fiancées or girlfriends of the perpetrators of the act. Sexual violence against girls (defilement) was most reported by the media (40.4%; *N* = 21) followed by other forms of abuse (25%; *N* = 13) such as beating, threatening, and sex trafficking. The other forms of VAWG covered by the media included rape, intimate partner homicide, other forms of murder/attempted murder, and outmoded cultural practices such as forced marriages and female circumcision. The actions taken against perpetrators included being jailed, arrested or standing trial in the court of law.

**Table 1:** Characteristics of VAWG

Violence domain	N (%)
Place of incident	
Urban	32 (74.0)
Rural	11 (26.0)
Type of violence:	
Sexual (defilement)	21 (40.4)
Sexual (rape)	8 (15.4)
Intimate partner homicide	7 (13.5)
Other murder (or attempted)	3 (5.8)
Other forms of abuse	13 (25.0)
Victims:	
Women	27 (51.9)
Girls	25 (48.1)
Perpetrators of violence:	
Men	38 (73.1)
Women	3 (5.8)
Parent	6 (11.5)
Society (Customary practices)	5 (9.6)
Immediate cause of the violence:	
Sexual desires	30 (68.2)
Infidelity	4 (9.1)
Financial	10 (22.7)
Action taken against perpetrator:	
Jailed	14 (29.2)
Arrested	10 (20.8)
Standing trial	12 (25.0)
Public censure	3(6.3)
Under police investigation	4 (8.3)
Not reported	5 (10.4)

Note: Due to missing data, variable attributes may not equal total articles examined. Other variable attributes may also exceed the total articles examined due to inclusion of multiple cases in a single article

### VAWG as a private/individual issue

This frame discusses how the news articles presented VAWG as an individual issue (episodic). The frame is grounded in how journalistic values of ‘timeliness’ and ‘personalisation’ resulted in VAWG coverage that tended to focus on the individual and hardly addressed these ‘event-based’ cases as a systematic social/public health problem. The current study found that the majority (92%; *N* = 44) of the articles framed VAWG as an individual incident with only 8% (N = 4) considering the broader social context of the incident. The four articles which focused on social context were media coverage of reports on interventions implemented by three non-governmental organisations, and one by the government sector ministry on Gender, Children and Social Protection. This organising frame is explained by two basic frames: sensational headlines and normalising the event as commonplace.

The headlines of the news encapsulating first-degree information were often used to project VAWG as an individual issue. Most of the headlines (90%; *N* = 43) made the perpetrators the subject of the story and the victims the object of the story. In some cases (38%; *N* = 18) the news headlines established the relationship between the perpetrators and the victims. Some of the headlines included: “Jealous man butchers wife” (*Myjoyonline.com*; 21–1-14); “Man beats wife to death over GH¢80 [$21]” (Myjoyonline.com; 23–01-2014); “Pastor rapes unconscious student after applying ‘anointing oil’” (*Daily Guide*; 10–04-2014); “She was abandoned, rejected, defiled and beaten but refused to die” (Myjoyonline.com; 01–10-14). Though the information captured in these headlines enrich the coverage with the context within which readers are able to identify the incident as VAWG rather than random violence, they are still problematic as journalists portrayed VAWG as an incident based issue perpetrated against women and female children by any type of offender. The initial paragraphs of these articles could have been used to situate the cases into the broader perspective but the analyses indicated that these were not done as journalists presented the stories as separate, discrete incidents. This reinforces the idea that VAWG is an isolated incident related only to the particular circumstances of those involved and unrelated to the larger structure of male dominance society. Also, articles framing VAWG as an individual issue in the headlines often described how the incident played out and who committed it but rarely delved into the broader question of ‘why’ except on a superficial level.

The analyses of the various texts indicated that in about 39 articles (81%), journalists used a frame that normalised the event as commonplace and therefore, individualised the actual acts against women and girls. In most cases, these news articles characterised the incidents as just another violent act against women which were news worthy. This was largely evident in the tones of journalists which were a complete re-echoing of court room proceedings with no additional input from the journalists to help put the issues into broader perspective. In these stories, the implications of the violent act committed against women and girls were not provided.

An instance of framing VAWG as commonplace included: “A 39-year-old electrician, Reuben Yartey, who sexually abused his 15- year- old daughter over a four-year period was on Wednesday sentenced to 25 years imprisonment by an Accra Circuit Court” (GNA; 05–03-14). In another article where a 12-year old girl was sexually abused by the father, the first two lines and the conclusion captured the story as follows: “A 30-year-old driver has been arrested for incest with his 12-year old biological daughter at Otoase near Nsawam in the Eastern Region. The police said Benjamin Tetteh [perpetrator], admitted to sexually abusing his daughter, a class two pupil on 10 different occasions and attributed his diabolical act to the devil…Tetteh was arrested and during interrogation admitted the crime and claimed it was the work of the devil.” (The Ghanaian Times, 30-05-2014) A journalist further reported in another article, ***“***The chief and elders of Wassa Saaman near Wassa Akropong in the Western Region have slapped a fine of two sheep and bottles of schnapps on a 35-year-old man for allegedly engaging in an abominable act that can incur the wrath of the gods in the area” (*Daily Guide*; 07–03-14). The use of language such as “four-year period”, “on 10 different occasions”, “slapped a fine” does seem to highlight the media’s characterisation of VAWG as one of many incidents of VAWG, and thus normalises the event as commonplace. These commonplace frames fail to critically examine the complexities surrounding VAWG to adequately inform the public about the social and health implications of the issue, and therefore constitute episodic framing of the issue.

### Victim blaming

This frame focuses on stories that blamed the victims for the occurrence of the violent act. The analyses indicated that victim blaming tactics were largely used across the articles (91%; *N* = 43). Both direct and indirect victim blaming tactics were found in this study. The three basic frames that explain the larger victim blaming frame are: media sources of information, exoneration of perpetrators and commodification of women.

#### Media sources of information

The news articles quoted sources such as perpetrators, family members and criminal justice officials blaming the victims. In one article, the journalist quoted a perpetrator who sexually abused 183 women within a year and used their vaginal fluids for money rituals: “Our women like money and I want them to know that not all that glitters is gold. They should be careful and not cheapen themselves for money” (*Myjoyonline.com*; 15-08-14). In another article, the brother of the victim stated the following about her sister who had been raped: “She was unwilling to report the incident until he (the brother) coaxed her to the police station where they both lodged a formal complaint”. In this same article, the journalist further quoted the brother as saying: “The investigator advised the lady to learn from what had happened, insisting her case was a feeble one and the Attorney General’s Department would not even consider it a case to be pursued” (*Myjoyonline.com*; 25-07-14). In another article, the journalist quoted the prosecuting attorney who blamed a 15-year old girl for failing to report previous sexual abuses from the father: “Prosecution contended that investigations revealed that the victim had suffered several sexual abuses for the past four years without complaint or reporting any of the cases” (GNA; 05-03-14). These articles use victim blaming tactics to highlight the victims’ failure to report the perpetrators. They are also suggestive of the fact that if the victims were a little more careful and vigilant, they could have prevented the act of violence. In most of these articles that used victim blaming language, the tone was not sympathetic to the victims as journalists relied solely on information from secondary sources particularly criminal justice officials.

#### Exoneration of perpetrators

Exoneration of perpetrators by focusing on the immediate cause of the violent act – the “why he [perpetrator] did it” aspect of news reporting also contributed to victim blaming. In this frame, the perpetrators actions were minimised and/or exonerated entirely. In an example of exonerating the perpetrator by focusing on why the incident occurred, one article opened as follows: “Feeling peeved for being jilted by his fiancé, a man decided to post naked pictures of his former lover on Facebook” (*Daily Graphic*; 01-23-14). This article captured a story involving a young woman who had called off a two-year old relationship with her fiancée. Without examining the relationship history of the victim and the perpetrator or providing any discussion on the emotional and psychological abuse meted out to the victim when the perpetrator [fiancée] posted the victim’s nude pictures on Facebook, the article concluded as follows: “According to the prosecution, Alibah [perpetrator] called the complainant [victim] and warned her to return to him or have her naked pictures posted on Facebook”. The concluding statement of this article clearly minimises the perpetrator's action. Similarly, the use of the word ‘jilted’ and the phrase ‘feeling peeved’ in the news articles are suggestive that the victim should have known the consequences of her action as expressed by the perpetrator, and taken appropriate measures to remedy the situation – thus blaming the victim.

In addition, media reports on sexual assault or harassment (intimate partner or rape) were found to be mainly centered on portraying women/girls as helpless victims who could not prevent the violent act from occurring. In some cases, the media defined in a realistic manner the violent acts against women and girls figuratively through the use of photos, as images to portray a real act or an actual fact.

Suspected infidelity (cheating and/or affairs) on the part of the victim or the perpetrator (9.1%; N = 4) was another factor that sought to exonerate the perpetrator and thus contributed to victim blaming. In instances where the cause of the violent act was said to be as a result of infidelity, this culminated in intimate partner homicide. For instance: “Justice Ampofo, a 38-year-old man last Saturday January 18 butchered his wife, Elizabeth Danso, 38, at Akyem Tafo in the Eastern Region over suspected infidelity… The suspect [perpetrator] attempted suicide by drinking poison shortly after the incident” (*Myjoyonline.com*; 21–1-14). This article mainly focused on describing how the suspected infidelity caused the perpetrator [described as a ‘jealous man’] to inflict cutlass wounds on the victim and afterwards decided to commit suicide. Across the 4 articles, infidelity was used as the causal argument for VAWG and therefore, turned attention away from the perpetrators’ culpability.

#### Commodification of women

Commodification of women contributed to victim blaming. In this frame, women were largely depicted as ‘commodities’ to be exchanged for money. In some cases where money was said to be the cause (22%; N = 10), journalists reported that this triggered off anger and argument resulting in the violent act. Journalists spent time describing and quoting sources of how refusal of women to return money to their perpetrators was a reason for the violent act suffered. This journalistic description largely projected women as commodities to be exchanged for money to satisfy the needs of perpetrators. For example, one article stated: “The Prosecutor said Akua refused to refund the money and Kwofie became unhappy, pushed her to the ground and subjected her to severe beatings with his legs and hands” (Myjoyonline.com; 23–01-2014). The case reported in this article was a gruesome murder of the victim over US$21. The entire story in the article indirectly blamed the victim for refusing to refund the money to the perpetrator – an action which infuriated the perpetrator resulting in an intimate partner homicide. Another article similarly sought to exonerate the perpetrator who attempted to murder his mother for money rituals: “The suspect had confessed to the act and added that life had been difficult for the family, which was why he wanted to sacrifice the mother who is currently sick to save the rest of the family from a biting poverty” (*Daily Guide*; 02–27-14). These individualised explanations of violence may exonerate the perpetrator by excusing the violent act committed, commodify women and thus trivialise VAWG.

In line with commodifying women, analysis of some stories in the news articles revealed that generally the perpetrators’ actions were masked by focusing on behaviours of the victims who were being used as ‘substitutes’ to satisfy the sexual urges of the perpetrators. In most cases (68%), journalists reported that the inability of male perpetrators to control their sexual urges resulted in rape, defilement, sexual harassment, physical abuse and in some cases murder. For instance, in one article, the journalist reported that the perpetrator defiled a 6 year old daughter because “He [perpetrator] was fed up with the boring sexual positions of the wife [victim]. To this end, any time Abudja [perpetrator] felt like having sex, he would just jump on the stepdaughter [victim] since she was conversant with the current sex styles” (*Daily Guide*; 07–03-14). In this article, the 6 year old girl [victim] is indirectly blamed for the defilement as she is reported as being a perfect ‘substitute’ to the mother by ‘being conversant with current sexual positions’ which could satisfy the sexual needs of her father [perpetrator].

## Discussion

The current study examined Ghanaian media coverage of VAWG and the implications of this for health promotion. With regard to the kind of information on VAWG provided by the media, the results indicate that the media covered areas such as the nature of violence, the victims and the perpetrators involved in the act, the immediate cause of the violence, and the actions taken against the perpetrators. These findings require further investigation in order to assess their contributions to the VAWG literature particularly in terms of how the provision of these information would shape the public’s perception of VAWG as a public health issue.

In relation to how the media framed VAWG, the findings point to dominant frames: victim blaming and episodic framing. First, taken together as a group, the analysis of VAWG coverage in Ghana showed increased used of victim blaming language than previous studies conducted in the US [24, 25, 28, 44] which similarly identified frames that blamed the victim and/or exonerated the perpetrator in relation to domestic violence. While journalists did not directly blame the victims in the current study, direct victim blaming tactics used in the secondary sources of the news articles to blame the victims included the suggestion that they had failed to take appropriate measures to protect themselves, suspected infidelity and not filing charges for abuse or not reporting previous violence. In reporting the behaviours of the victims before, during and after the incidents, blame is shifted either directly or indirectly from the perpetrators to the victims. The indirect victim blaming tactics included highlighting the perpetrators’ emotional problems and the tone of the article. These findings corroborate the findings of Sutherland et al.’s [45] review of studies on news media coverage of violence against women which showed that the media’s usage of information from secondary sources resulted in either direct or indirect victim blaming. The findings further align with Breen et al.’s [46] analysis of Australian Journalists discursive practices in reporting rape and Bonnes’ [47] analysis of newspaper coverage of rape in South Africa which all concluded that lexical features and emphasis on the perpetrators’ feelings contribute to minimising the harm caused to the victims, and result in victim blaming. The findings are also similar to those of Gillespie et al. [25] who found that the news media in the US largely framed femicides as commonplace, downplayed the issues through victim blaming, and failed to hold the perpetrators responsible for femicide cases.

Specifically, in the present study, women and girls were mainly framed as victims across all news articles. These frames maintain a critical disconnection between VAWG presented as isolated, individual cases, and VAWG as a broader social problem. Framing victims of VAWG as helpless individuals may similarly result in victim blaming. When individuals are made to feel responsible for their own victimization, they are less likely to seek for help and the probability for maintaining social tolerance for the action increases [48, 49].

A key feature of the 48 news articles included in the analyses was that the framing of VAWG was episodic in nature as the acts of violence perpetrated against women and girls were presented as individual cases without reference to the wider social contexts within which they occurred. For all forms of violence reported in the news articles, a pattern emerged where the act was framed as individual aberration, a reflection of conduct by one individual against another individual (mostly males against females), and in some rape cases several individuals against another was observed. This is consistent with a two-year study of newspapers and violence against women in the US by Carlyle, Slater and Chakroff [29] which found that 88.3% of stories in their sample used episodic framing. This finding also aligns with several studies examining VAWG which suggest that media portrayal is skewed towards episodic framing rather than thematic framing [25, 28, 44, 45, 49]. This may reinforce in the minds of the public the idea that VAWG is an interpersonal rather than a public health issue.

For several cases of VAWG in the current study, frames were not mutually exclusive. For articles employing episodic frames, the commonplace frame was also used to describe cases as another form of VAWG. These findings support Gillespie et al.’s [25] study on media framing of domestic violence in North Carolina which revealed that domestic violence was framed as episodic with commonplace frame. In the current study, both state owned media and private media outlets used episodic framing. This is contrary to Consalvo’s [50] study which revealed that minority presses in Seattle linked an individual’s case of domestic violence murder to the larger problems of domestic violence and violence in general, and further encouraged the readers to see the problem as a social issue that must be addressed.

Similarly, as reported in previous research, the news headlines largely presented the victims in the context of a familial relationship (e.g. wife, fiancé, daughter, or mother) while the perpetrators who were mostly males were described in terms of their occupations. This finding is in line with Evans’ [51] study which examined media coverage of domestic violence in Australia and found that in headlines, female victims tended to be portrayed in terms of their familial relationships (i.e. ‘wife’ or ‘mother’), while male assailants were most often described in terms of their professional background (e.g. teacher, pastor, police, etc.) and never as ‘husbands’. This clearly supports the gender symmetry debate where women are regarded as subordinate to men or as having stereotype roles thus perpetuating VAWG [52].

Unlike Bullock’s [14] study which examined domestic violence in Utah where most articles (95.3%; *N* = 43) used words such as ‘domestic violence’ or ‘abuse’, in the current study, the terms ‘violence against women/girls’ or ‘abuse’ was never used in the articles. Rather, words that denoted the specific form of violence which had occurred were used: rape, defilement and murder. This might have contributed to the journalists’ inability to thematically frame VAWG in the social context to better educate the public and also get the issue unto the political agenda. Failing to frame VAWG as a systematic social/public health problem is worrying as it shifts the responsibility for solving the problem from society to the individual victim and the perpetrator. Similarly, episodic reportage of VAWG misinforms the public that VAWG is an isolated incident, and the public may fail to understand the prevalence of VAWG both as an ongoing problem for many victims and as a social problem which needs to be tacked head-on [29]. Furthermore, in Ghana and many other low-and middle-income countries, where the public already perceives VAWG as a private/individual issue rather than a social/public health problem, efforts towards changing this ingrained societal belief is thwarted if such normative beliefs that trivialise VAWG are sustained and reproduced by news coverage that thrives on ideologies established decades ago. Again, episodic framing of the issue is counterproductive to public health interventions and academic work that have approached VAWG as a social and public health issue rooted in social norms, gender inequalities, and patriarchal society that nurture VAWG. Ideal news coverage of VAWG should utilise the perspectives of VAWG experts and frame the issue thematically.

### Implications for health promotion

First, the findings suggest that VAWG in Ghana receives limited coverage in the media which emphasises the private nature of the issue. As we have shown, only 52 cases of VAWG were published by the top media outlets in the year 2014 although available statistics for the period indicated a high prevalence of VAWG [3]. As noted by Entman [15], news media framing influences both political decision and public opinion. Therefore, the lack of coverage by the key news media outlets points to a missed window of opportunity from the point of view of agenda setting and framing, and further downplays VAWG as topic worthy of attention, and collective action.

Second, the results raise questions about how well the media inform the public about VAWG. It appears that the public is being informed that VAWG is an individual affair so people should take responsibility for it. This finding points to a need for collaboration among health promoters and other experts working in the field, the judicial service, and the media to ensure objective and comprehensive news coverage and dissemination of information on VAWG. This collaboration can for example, result in the development of relevant media guidelines to promote accurate, sensitive and responsible coverage of VAWG within its social context as well as training journalists on appropriate framing of VAWG in the media to contribute to prevention efforts. As observed by Richards, Gillespie and Smith [53], studies of this nature examining media portrayal of VAWG are useful in developing recommendations for effective media coverage of VAWG to avoid contagion effects. Sutherland et al. [54] add that to promote broader media reportage on VAWG, there is the need to complement media guidelines with approaches that engage with the media through consultation and training. Similarly, it is imperative that health promoters and public health experts become conscious of how to effectively respond to incidents of VAWG in ways that are media friendly. For instance, effective and timely use of press releases by the Ghana Health Service and the Ministry of Gender, Children and Social Protection about new interventions and resources available for prevention and control of VAWG would go a long way to help keep the public well informed.

Also, the analysis of news articles in this study revealed that omission was the main strategy often employed by journalists which contributed to episodic framing and victim blaming. Apart from the age of the victims which most of the news articles provided (57%; *N* = 26), other key features of journalistic description such as the level of education, occupation, religious characterisation, ethnicity and others were disregarded. Similarly, all the news articles failed to provide information on support services that had been put in place for the victims. Richards, Kirkland and Smith [28] note that by using sources such as victim advocates, victims and their families may be pointed to resources available to assist them as well as victims being encouraged to seek help. This implies that the news media should consider a greater use of VAWG advocates and professionals in their reportage as this may help contextualise VAWG, explain the rationale for such incidents, and bring to the fore support services available to victims. Also, all the news articles in the present study did not discuss the health implications of the acts of violence meted out to the victims. It may be that these omissions are a result of non-utilisation of appropriate advocates and authorities for VAWG as news sources. Another possible argument justifying the absence of basic descriptive features of the victims and the perpetrators may be that it is ‘not the work’ of the news media to educate the public in such a manner. However, for effective targeting and development of health promotion interventions, it is important that news media coverage on the issue moves beyond just identification of the victims and the perpetrators to providing key information such as education, risk behaviours (e.g. use of alcohol, drugs or smoking) and any past history of abuse, or a history of being a victim or a perpetrator.

The media play an important role in the prevention of VAWG. Therefore, it is important that media reportage on the issue moves away from episodic to thematic framing so as to provide a broader context that would inform public opinion and engender policy response. In effect, for the media in Ghana to contribute to the prevention of VAWG, there is the need for their news coverage to focus on social construction of the issue, challenge gender/social norms that are inimical to prevention efforts, raise awareness about support services available to victims, and also elicit political will for investment of resources for health promotion interventions focusing on prevention of VAWG. In this regard, the health promotion department of the Ghana Health Service, the Ministry of Gender, Children and Social Protection, the Gender Centre, and other civil society organisations working with women and children should consider partnering the media in Ghana to strengthen the capacity of journalists in VAWG education and prevention.

### Limitations of the study

The sample of news reports for this study was limited to those published in English and it is possible that by not including non-English media sources, we may be missing out on framing that is done in those news items. Similarly, while we acknowledge that VAWG can take on many forms, our sample only includes coverage of physical violence. This narrowing of scope of VAWG constitutes a limitation to the study as the Vienna Violence Against Women Declaration does include non-physical violence as well. As noted before, our comprehensive search for news articles from the six mainstream media outlets found a coverage of 52 VAWG cases out of 19,286 cases reported by the DOVVSU in 2014. That is, the news coverage reported here do not address the universe of VAWG cases in Ghana as a greater percentage of VAWG cases remain uncovered by the mainstream media. Notwithstanding these limitations, the external validity of the findings is strong as the findings are largely consistent with those reported in the extant literature. The findings thus provide useful insights to policy makers, health promoters, journalists and VAWG advocates on the need to optimise news media coverage of VAWG.

## Conclusions

The results from this study contribute to the limited literature on VAWG in non-Western, low-and middle-income country context. By providing a unique study of how the news media frame VAWG in Ghana, the current study has demonstrated that a lot more needs to be done by the media particularly those in low-and middle-income countries in terms of their coverage of VAWG for effective prevention of the problem.

Contrary to findings of studies from the US where to some extent the media were said to provide a wide coverage and also run stories on VAWG over a long period of time [14, 24, 50], the media in Ghana were found to report on such issues only once, and therefore, failed to adequately push the issue on the policy agenda and/or adequately inform the public about the issue. Given the ability of the media to educate and galvanise public health efforts, for broader coverage of VAWG, there is the need to cite experts, provide statistics, and include contact information for improved awareness creation and prevention of VAWG.

To a larger extent, the current study confirms the findings of Bullock [14], Bullock and Cubert [24], Gillespie et al. [25], Richards, Kirkland and Smith [28], Richards, Gillespie and Smith [53] (all in the US), Fairbairn and Dawson [32] (Canada), Mason and Monckton-Smith [33] (UK), and Morgan and Politoff’s [31] (Australia) studies that the media largely blame victims and frame VAWG episodically. It can therefore, be argued here that the media in both high-income and low-and middle-income countries equally fail to use their frame power to project VAWG as broader social and public health issue. The recognition of VAWG as a shared social problem can lead to better public policy initiatives, including resources for victims. This is where the media through responsible framing have the unique ability to shape public opinion concerning VAWG as well as society’s role in prevention efforts. Effective collaboration between the media and health promoters and other advocates is however, critical to the prevention and/or control of VAWG.

Clearly, this study has highlighted how patterns in coverage about VAWG in Ghana prevent the public from gaining meaningful and important information about VAWG as a public health issue. It is our hope that through this research, best practices for media coverage on VAWG will be developed, disseminated, and implemented on a national level. Future research should critically examine how media reportage on VAWG aligns with the epidemiological data on the issue. Further research is also required on how media coverage of the issue informs the public’s perspective about the issue and the use of available services to prevent VAWG.

## Abbreviations

DOVVSU: Domestic Violence and Victim Support Unit of the Ghana Police Service
GNA: Ghana News Agency
NHIS: National Health Insurance Scheme
UK: United Kingdom
US: United States
VAWG: Violence against women and girls

## Footnotes

1 In Ghana, the Criminal Code amended by Act 458 section 2 (a) defines rape as the carnal knowledge of a female 16 years and above without her consent while defilement is the carnal knowledge of any female under 14 years of age, whether with or without her consent.

2 A total of 6380 more cases of VAWG were reported in 2014 as compared to 2011 (DOVVSU, 2014).

3 According to the 2010 census report, about one-fifth (20.1%) of the population in Ghana can read and write in the English language (Ghana Statistical Service, 2013).
